# The RNA-binding protein QKI suppresses tumorigenesis of clear cell renal cell carcinoma by regulating the expression of HIF-1α

**DOI:** 10.7150/jca.36083

**Published:** 2020-01-01

**Authors:** Fei Shi, Di Wei, Zheng Zhu, Fei Yan, Fuli Wang, Keke Zhang, Xi'an Li, Yu Zheng, Jiarui Yuan, Zifan Lu, Jianlin Yuan

**Affiliations:** 1 Department of Urology, Xijing Hospital, Fourth Military Medical University, Xi'an710032, China; 2 Department of Urology, The 201 Military Hospital, Liaoyang 111000, China; 3 State Key Laboratory of Cancer Biology, Department of Pharmacogenomics, Fourth Military Medical University, Xi'an710032, China.; 4 School of Medicine, St. George's University, Grenada.

**Keywords:** QKI, tumor suppressor, HIF-1α, survival, biomarker background

## Abstract

**Backgrounds**: A number of genetic and biological phenomena imply that tumorigenesis of clear cell renal cell carcinoma (ccRCC) is highly correlated with hypoxia-induced factor-1a (HIF-1α). Recently, research focusing on the post-transcriptional regulation of HIF-1α has provided a new perspective for ccRCC therapy. In this study, we observed the expression pattern of the RNA-binding protein QKI, which could regulate HIF expression in ccRCC both *in vitro* and *in vivo*.

**Methods**: Tissue microarraywas subjected to immunohistochemistry and tumour cell lines and nude mice were used for *in vitro* and *in vivo* assays. QKI overexpression or knockdown was assessed in renal cancer cells.

**Results**: The overexpression of QKI inhibited the proliferation of the 786-0 and caki-1 cells, blocked the cells' entry into the S phase, and promoted apoptosis. In ectopic-implantation nude mice model, QKI depletion significantly increased tumor sizes and initiation rates. Tissue microarrays showed that the expression of QKI genes, and especially QKI-6, was significantly decreased in tumor tissues compared with these in normal kidney tissues. Moreover, decreased QKI expression was closely correlated with high tumor grade, poor differentiation, and poor survival.

**Conclusions**: QKI may be useful as a novel, independent diagnostic and biological marker for ccRCC.

## Introduction

The morbidity of ccRCC has increased, which makes ccRCC one of the most notorious diseases threatening the health of the general population. Renal-cell carcinoma (RCC) accounts for 1.9 % of all cancers is one of the most common type of cancers in adults and 1-3. Von Hippel-Lindau (VHL), a tumor suppressor gene, frequently shows a somatic mutation in numerous RCC patients. 2, 4. Meanwhile, the VHL gene encoded protein pVHL is a crucial regulator of the oxygen sensing process, involving the transcription factor HIFα 2, 5. pVHL is part of the recognition component of a ubiquitin ligase complex that targets HIF-α subunits for degradation under normoxi conditions - a process that is inhibited under hypoxic conditions (typically < 5 % O_2_) by several mechanisms. What is more, pVHL can affect fibronectin assembly 6. HIF is a heterodimeric transcription factor activating the expression of various genes involving angiogenesis, erythropoiesis, energy metabolism, apoptosis, and/or proliferation in response to hypoxia conditions. Under normoxia conditions, pVHL inhibits HIF activity by targeting its α-subunits for polyubiquitination and proteasomal degradation 7-11.

RNA-binding proteins and microRNAs are key regulators in cell biology, by regulating the RNA stability, transcriptional efficiency, translocation and alternative splicing. Deficiency of mRNA regulation is closely related to cancer as well as many other human diseases 12. QKI-5 binds to QKI responsive elements (QRE) in their 3′UTRs region containing NACUAAY-N(1-20)-UAAY sequences to regulate the localization, stability, and translational efficiency of its target mRNAs and then to modulate physiological and pathological processes^13^. Latest studies have demonstrated that abnormal expression of QKI is associated with the progression of a number of different human cancers, such as prostate, colon, gastrointestinal, and oral cancer 14-17.These lines of evidence indicate that QKI plays a role as a tumor suppressor gene in a variety of cancers.

Although it has recently been proven that QKI-5 inhibits ccRCC proliferation through the RAS-MAPK signal pathway, we have differentiated the mechanism by which QKI inhibits ccRCC from its roles in metabolism and angiogenesis. Above all, with new insights into the different functions of QKI, we conducted a series of interesting tests aiming to improve the diagnosis, prognosis and therapy of ccRCC. We tested the relationship between renal cell carcinoma and expression of QKI in the following aspects:

(1) The expression of QKI in renal cell carcinoma and adjacent normal tissue;

(2) The correlation of QKI with the degree of differentiation, degree of invasion, TNM grading, age, tumor size and survival rates of patients with renal cell carcinoma; and

(3) How the expression of QKI influences the biological behavior of the cells and tumors both *in vitro* and *in vivo*. In view of our findings, we have come to the conclusion that QKI inhibits the development of renal cell carcinoma by decreasing the expression of HIF-1α. Moreover, QKI maybe an independent factor that influences the prognosis of renal cell carcinoma, and therefore may serve as a potential diagnostic and therapeutic target.

## Methods

### Tissue sample collection and study cohort

This study was approved by the ethics committee of the Fourth Military Medical University (Xi'an, PR China). We used tissue microarray technology to analyze samples from 161 participants who were diagnosed with clear cell renal cell carcinoma between July 2007 and February 2008. Patients, who received treatment before surgery, including partial or radical nephrectomy, were excluded. A part of the patients (129) went through follow-up visits up to September 2012. The cancer tissues and the matched adjacent normal tissues of every participant had been reviewed and confirmed by the Department of Pathology, Xijing Hospital, China. Pathological information was collected from the clinical patient database, and the information was blinded for the study physicians who reviewed all the records of ccRCC and recorded the data into the database.

### Immunohistochemistry

The QKI antibody was purchased from Sigma Biotechnology, America, and the assay was conducted according to the manufacturer's instructions. Tissue array was fixed in 10% neutralized formalin and was embedded in paraffin blocks. Tissue arrays (4 μm) were prepared for H&E staining and also for immunohistochemical examination. For immunohistochemical processing, the endogenous peroxidases were blocked using 0.75% H_2_O_2_ in phosphate-buffered saline (PBS) for 50 min, followed by incubation in 5 % bovine serum albumin blocking buffer. The tissue sections were incubated with the primary anti-QKI antibody (1:300; sigma, America) for 24 h, 4 °C. Immunodetection was performed using a 3-step protocol, with a streptavidin-horseradish peroxidase complex and visualized using 3, 3-diaminobenzidine.

### Total survival time

The final follow-up was completed in December 2012, with around 5 years of monitoring for each patient. The follow-ups were conducted from the date of surgery until death or the December 2012 deadline. The doctor responsible for recording the follow-up information did not have access to the clinical pathological database and the QKI immunohistochemistry results.

### Cell culture and transfection

The 786-0 and caki-1cell lines were purchased from American Type Culture Collection (ATCC, Manassas, VA, USA) used to model renal cell carcinoma, and the HEK293 (ATCC) cell line was purchased from Chinese Academy of Sciences and used to model normal kidney tissue. The cells were cultured in RPMI 1640 medium (HyClone, USA) supplemented with 10% FBS(fetal bovine serum; Gibco) and 0.1 % penicillin-streptomycin (1:1000), seeded into 25 cm^2^ plastic cell culture flasks (Corning), and grown at 37 °C in an atmosphere comprising 5 % CO_2_and 21 % O_2_. The cells were maintained as monolayers in 60 cm^2^ treated tissue culture flasks (Corning,) at 37 °C in a humidified atmosphere comprising 5 % CO_2_. Vector transfection was conducted using the FuGENE 6 kit (Roche) in accordance with the manufacturer's instructions. For the hypoxia treatment, cells were cultured under hypoxic conditions (3 % O_2_, 5 % CO_2_ and 92 % N_2_) and harvested at 24 h after transfection.

### Protein extraction and western blotting

RIPA buffer (Biotime, China) was used to extract total cellular protein from the two renal cell carcinoma cell lines, renal cell carcinoma tissues and adjacent normal tissues. The BCA assay was conducted to quantify the amount of protein, and 60 μg of protein from each sample was subjected to SDS-PAGE on 10 % polyacrylamide gels, and transferred to nitrocellulose membranes. The membranes were incubated with a primary antibody raised against QKI (1:1000; sigma) and other regulatory proteins (Abcam, Cell Signaling) overnight at 4 °C. After the membranes were washed with TBST, they were incubated with an IgG-IRDye TM800CW (Abcam, USA) fluorescently-labeled secondary antibody (diluted 1:10000 in TBS) for 40 minutes at 37 °C. The protein bands were visualized using an Odyssey Infrared Imaging Laser scanning imaging system.

### Real-time PCR

Cells that underwent the indicated treatments were harvested for RNA isolation using the Trizol reagent (Invitrogen, USA) according to manufacturer's instructions, and the RNA concentration was measured using a UV spectrophotometer. One microgram of total RNA was used as template for reverse transcription in accordance with the instructions of the M-MLV assay kit (Invitrogen). The primer sequences used in this study are shown in Table S1. PCR reactions (10 μl) were performed in a Bio-Rad PCR amplifier. The real-time PCR reaction system included 5 μl of SYBR Premix Ex Taq (Thermo Fisher, USA), 0.5 μl of forward primer (10 pmol/μl), 0.5 μl of reverse primer (10 pmol/μl), 1 μl of cDNA template (500 ng/μl), and 3 μl of ultrapure H_2_O.

The amplification conditions comprised 40 cycles at 95 °C for 5 s and 65 °C for 30 s. The results were analyzed by the Bio-Rad CFX Manager software.

### Flow cytometric analysis

To analyze the cell cycle distribution, cells were washed twice in PBS and fixed for at least 2 h in 300 μl PBS and 700 μl ethanol. The resulting fixed cells were spun down gently in 200 μl of extraction buffer (0.1 % Triton X-100, 45 mM Na_2_HPO_4_ and 2.5 mM sodium citrate) at 37 °C for 20 min and then re -suspended in PBS containing 40 mg/ml PI (propidium iodide; BD), 0.1 mg/ml RNase (Sigma, USA) and 0.1% Triton X-100 (sigma, USA) at 37°C for 30 min in the dark. Cell cycle distribution was detected by FACS on an instrument (Becton-Dickinson, USA).

### MTT assay

The MTT test was used to assess cell proliferation. Briefly, cells were seeded at a density of 2 × 10 ^3^ cells/well in 96-well plates comprising 100 µL of medium (HyClone, USA) per well. After 24, 48, 72, 96, 118 hours of incubation, 20 μl/well of a 5 mg/ml 3-(4, 5-methylthiazol-2-yl)-2, 5-diphenyl-tetrazolium bromide (MTT;) in 200ul solution was added and incubated for 4 hours at 37 °C. Next, the culture medium was removed, and 150 μL of DMSO was added. After 15 min of gentle shaking, the absorption at 490 nm was measured on a multi-well plate reader. Cell growth curves were determined according to the average absorption values.

### Lentiviral transfection

We selected the 786-0 cell line for this series of experiments. 1 × 10 ^5^ cells per well were seeded in 6-well plates (Corning). The culture medium was removed when the cells were 75 % confluent. One milliliter of lentiviral particles targeting sh-QKI was added. Scrambled lentiviral particles were added as controls. Afterwards, the cells were cultured at 37 °C in an atmosphere comprising 5 % CO_2_. Eight hours post-infection, the lentiviral particles were removed, and the cells were switched to regular culture medium for 24 hours. Subsequently, 1640 medium (HyClone, USA) with 5 µg/ml puromycin was used to culture the cells for another 24 hours. After 3 days, lentivirus-positive cells were selected based on conditions above. Western blot analysis was used to evaluate the efficiency of infection. The positive clones were selected for animal experiments after seven days.

### Enzyme-linked immunosorbent assay (ELISA)

The human VEGF ELISA KIT (Elabscience, China) was used to investigate the VEGF expression profile of human renal cell carcinomas. 786-0 and caki-1 cells were cultured for 24 hours at 37 °C in an atmosphere comprising 5% CO_2_ after transfection with plasmids. We collected the supernatants after culturing in serum-free 1640 medium for 24 hours. The ELISA was conducted in accordance with the manufacturer's instructions. The absorption values were measured at 450 nm on a multi-well plate reader.

### Animal Experiments

Male nude mice (4-6 weeks old) were obtained from the Experimental Animal Center of the Fourth Military Medical University (Xi'an, PR China). All animal experiments were performed in accordance with the Animal Care and Use Committee of the Fourth Military Medical University (Xi'an, PR China). Animals were looked after very well during experiment process. The 786-0 cells were suspended in sterile PBS and injected subcutaneously into the dorsal area of the nude mice. The size and incidence of subcutaneous tumors was recorded every 3 days. Tumor volumes were calculated according to the formula V (mm^3^) = width^2^ (mm^2^) × length (mm) / 2. At the indicated time points, the mice were sacrificed after barbiturate overdose by a properly trained person via cervical dislocation, and the cadavers were dissected for the evaluation of tumor size, and immunostaining.

### Immunofluorescence and determination of blood-vessel density

The effect of cell transplantation on neovascularization was assessed by counting the number of blood vessels, microvessel count (MVC), under a light microscope at 200× magnification. Tumor masses were obtained from nude mice at day 33. Frozen sections with 5 µm thickness were prepared from each specimen and were stained for CD31 (Servicebio, China) to detect blood vessels. Three fields from 2 tumor masses of each mouse were analyzed.

### Statistical analysis

Statistical analysis was performed using SPSS 17.0 software. Pearson's chi-square (χ^2^) test was used for QKI expression measurements. The Kaplan-Meier estimator was used to assess the survival rates. Statistical significance was evaluated using the means ± SD by single-factor analysis of variance (one-way ANOVA). Differences were considered statistically significant at *P* < 0.05.

## Results

**Figure 1:**
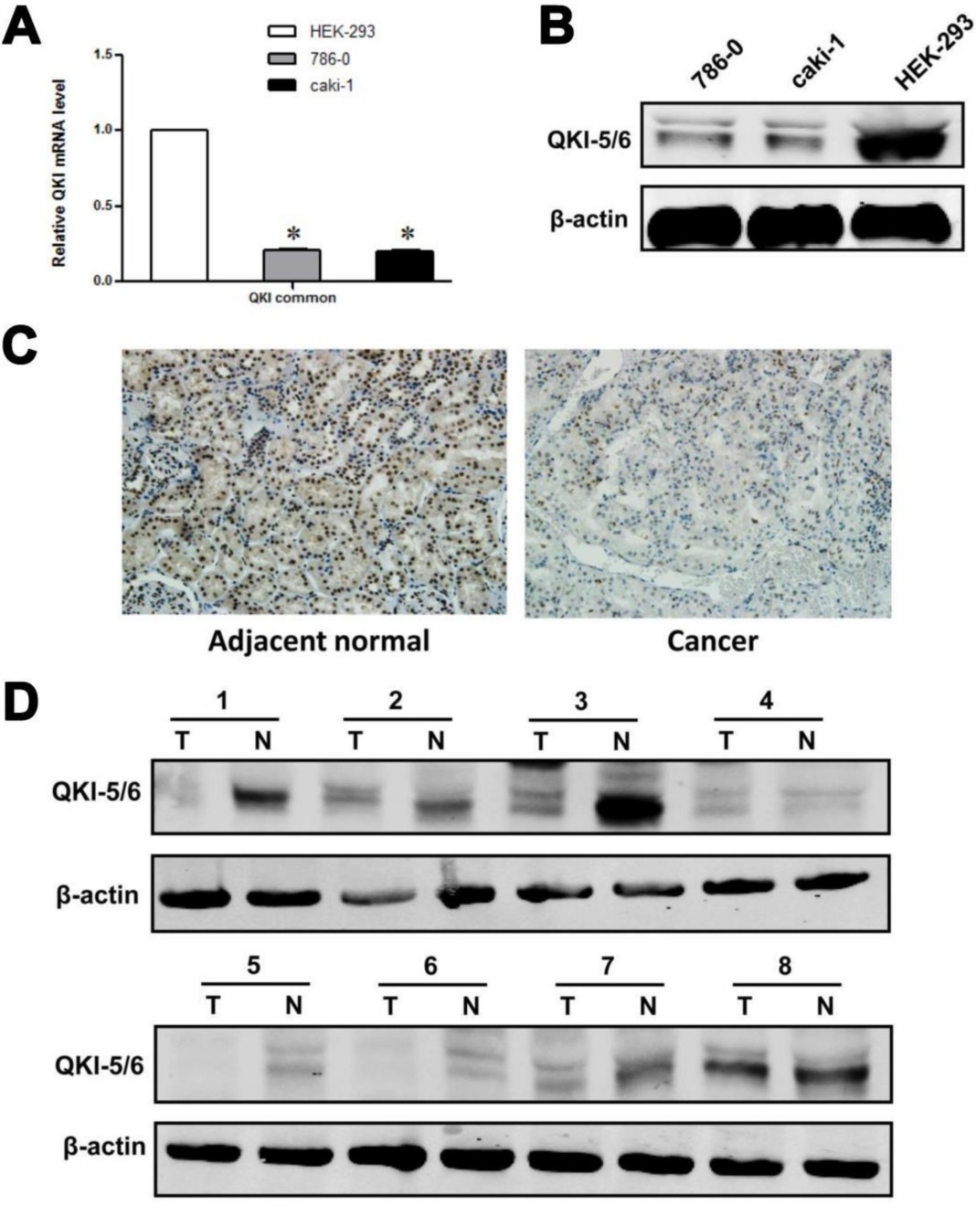
**QKI protein expression in ccRcc cell lines and tissue samples. (A)** mRNA levels of QKI in HEK-293,786-0, and caki-1 cell lines were analysis by RT-PCR. Results were normalized to β-actin mRNA. Data are shown as mean ± SD from 3 independent experiments. **(B)** Protein levels of QKI in the above cell lines were detected by western blot, and β-actin served as an internal control to ensure equal loading. **(C)** The QKI protein expression in ccRcc tissues detected by immunohistochemistry. The QKI protein expression levels were lower in most cancerous tissues than in the matched adjacent normal kidney tissues (200×). Data presented are representative of all samples. **(D)** Western blot analysis of the QKI expression in fresh clinical samples. The differences in the protein expression levels between the ccRcc and adjacent normal tissues were significant. Data presented are representative of all samples. The data are presented as the mean ± SD an d one-way ANOVA analysis for three independent experiments. * *P* < 0.05.

Our initial efforts focused on investigating the relationship between QKI and tumor growth. As an RNA-binding protein, QKI may function by modulating the expression of its target mRNAs at the posttranscriptional level. We noted that HIF-1α, which is highly expressed in VHL-mutated renal cancer cells, acts as a downstream effector of QKI. Analysis of QKI expression by western blotting and RT-PCR in two ccRCC cell lines and one normal kidney cell line showed that QKI expression was significantly lower under pathological conditions than in the normal cell line (**Fig. 1A and B**).To investigate the role of QKI in the initiation and progression of ccRCC, western blot analysis and immunohistochemistry were conducted to analyze the expression of QKI in the ccRCC tumor mass and matched adjacent normal tissues of 161 patients. As shown in **Table 1**, QKI expression was evident in 97.5% (157/161) of the matched adjacent kidney tissue samples, which represented a significantly higher percentage than in the QKI-positive samples of clear cell renal cell carcinoma (74.5% [120/161], χ2 = 20.99, *P* < 0.005). According to immunohistochemistry analysis, the expression of QKI in the nuclei and cytoplasm of ccRCC and matched adjacent normal tissues was completely different. The percentage of nucleus-positive samples of ccRCC was 74.5% (120/161), and the percentage of nucleus-positive samples of matched adjacent normal tissues was 75.7% (122/161). Accordingly, there was no statistical significance between ccRCC and normal kidney tissues χ2 =0.07, *P* > 0.05, as shown in **Tables 2 and 3**. By contrast, the percentage of cytoplasm-positive tissues in ccRCC was 6.2% (10/161),whereas the percentage of nuclear-positive tissues of matched adjacent normal tissues was77.6 % (125/161), which represented a highly statistically significant difference between ccRCC and normal kidney tissues (*χ2* = 84.34, *P* < 0.001), as shown in** Table 3**.

We further used western blotting and immunohistochemistry to illuminate the differences in the levels of relevant proteins in ccRCC and matched adjacent normal kidney tissues. The results of the western blot and immunohistochemistry analyses were similar, and representative data are shown in **Fig. 1C and D**.

These data demonstrated a significant reduction of QKI protein expression in clear cell renal cell carcinoma, which suggested that QKI may act as a tumor suppressor gene in ccRCC.

### QKI affects the proliferation of ccRCC cells

**Figure 2:**
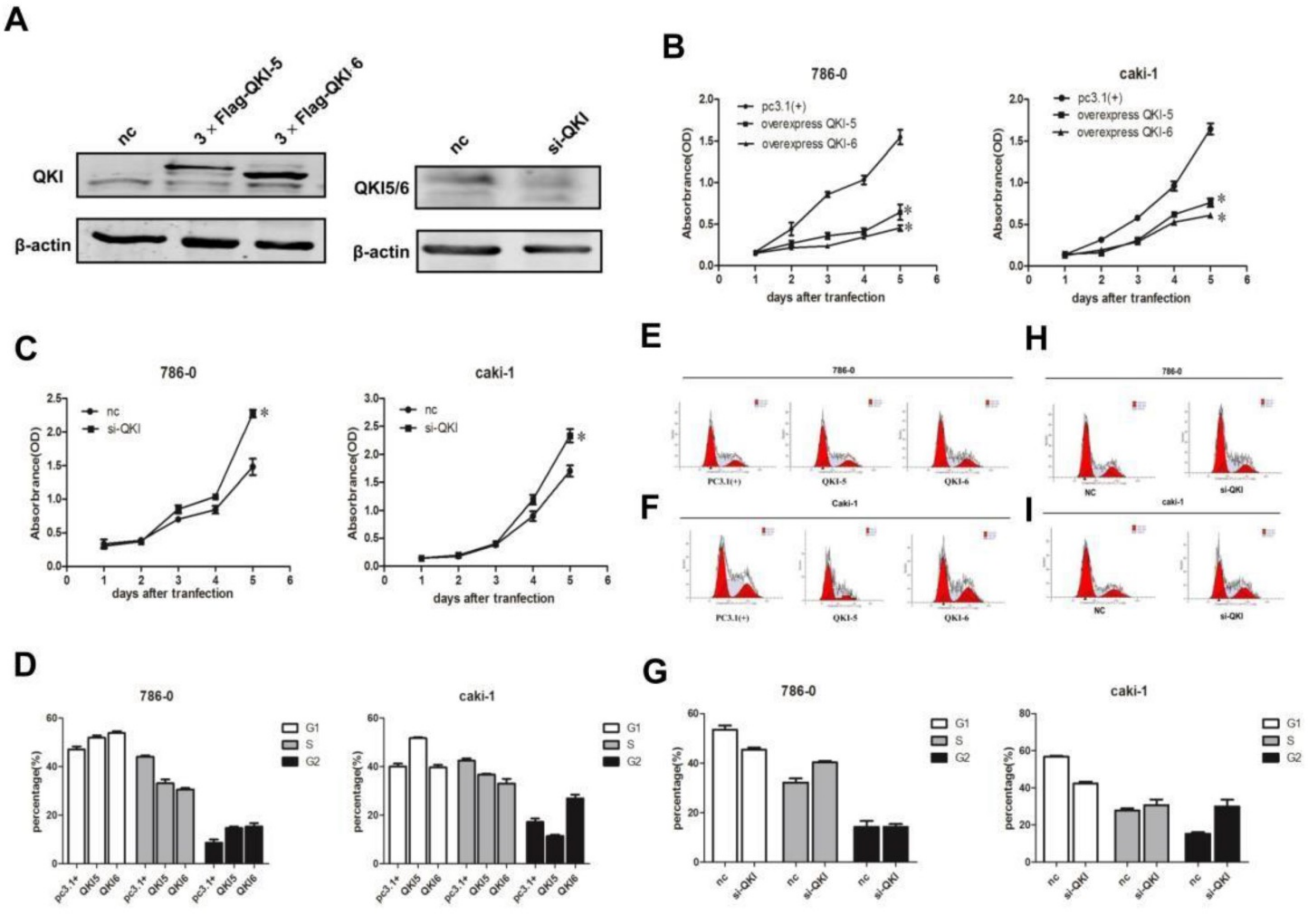
**Effects of QKI on ccRcc cell growth, cell cycle. (A)** The efficiency of QKI over-expression (left panel) and knockdown (right panel) in 786-0 cell line. **(B-E)** The cell growth curves are based on the average absorbance values (*n* = 6) detected with an auto-kinetic enzyme scaling meter using the MTT method. The cell growth curves showed that over-expression of QKI **(B)** significantly inhibited the growth of 786-0 and caki-1 cells. all assays were performed three independent times. The cell growth curve showed that knockdown of QKI **(C)** could promote the growth of 786-0 and caki-1 cells. **(D-F)** The 786-0 and caki-1 cells transfected with QKI plasmid were more easily arrested in the G0/G1 phase. **(G-I)** Knocking down QKI in 786-0 and caki-1 cells were easily promote cells entered into the s phase of the cell cycle.

In order to investigate the effects of QKI on the biological behavior of cancer cells, we overexpressed and knocked down QKI in the 786-0 and caki-1 cell lines, and verified the efficiency of the expression manipulation in the 786-0 cell line (**Fig. 2A**). To evaluate the extent of the QKI up- and downregulation, we utilized western blotting and RT-PCR. Transfection with QKI-modulating vectors was used to both upregulate and downregulate its expression both in 786-0 and caki-1 cells. Cell proliferation curves showed that the overexpression of QKI inhibited the proliferation of 786-0 and caki-1 cells to an increasing degree over time (**Fig. 2B**), while the downregulation of QKI resulted in elevated proliferation of both 786-0 and caki-1 cell (**Fig. 2C**). FCM analysis showed that the overexpression of QKI in 786-0 and caki-1 cells created a block in the G0/G1 phase which was more prominent than in the control group (**Fig. 2D-F**). Conversely, knockdown of QKI in the 786-0 and caki-1 cells promoted their entry into the S phase of the cell cycle (**Fig. 2G-I**).

### QKI affects the apoptosis of ccRCC cells

**Figure 3:**
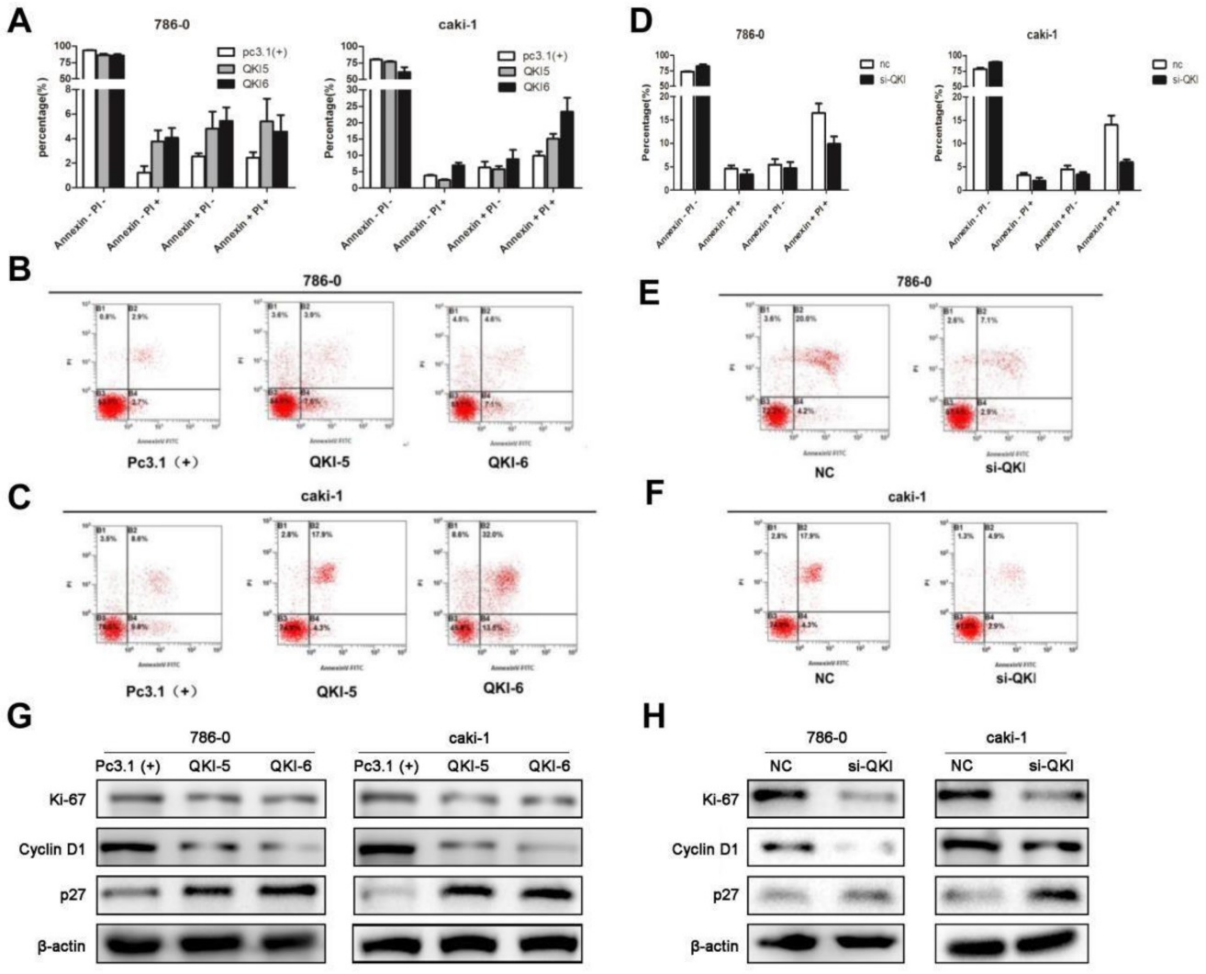
**QKI affects the apoptosis of ccRCC cells. (A-C)** The percentage of apoptotic cells in the QKI group was increased compared with the other two groups. **(D-F)** The percentage of apoptotic cells in the QKI knock-down group was decreased compared with the other two groups. **(G)** The effect of QKI overexpression on the G1 phase and the proliferation regulators as analyzed by western blot. Western blot analysis showed that in the QKI overexpressing 786-0 and caki-1 cells, the cyclin D1 and Ki-67 expression levels were downregulated and that the P27 expression level was upregulated. **(H)**The effect of QKI silencing on the G1 phase and the proliferation regulators as analyzed by western blot. Western blot analysis showed that in the QKI silencing 786-0 and caki-1 cells, the cyclin D1 and Ki-67 expression levels were upregulated and that the P27 expression level was downregulated. Data presented are representative of three individual experiments.

As cell survival relies on proliferation and apoptosis, we next explored the relationship between QKI and apoptosis. The percentage of apoptotic cells in the QKI-overexpressing group was significantly higher than in the pc(3.1+)group (**Fig. 3A-C**), while the percentage of apoptotic cells in the QKI knockdown group was significantly lower than in the negative control group (**Fig. 3D-F**).

Our previous study demonstrated that QKI was positively correlated with cell apoptosis and negatively correlated with cell proliferation, we then examined the relationship between QKI and cell cycle markers. Western blot analysis showed that in the QKI-overexpressing 786-0 and caki-1 cells, the expression level of cyclin D1was downregulated and the expression levels of P27 and Ki-67 were up-regulated (**Fig. 3G**).Conversely, in the QKI knockdown groups of both 786-0 and caki-1 cells, the CyclinD1 expression level was upregulated, and the P27 and Ki-67 expression levels were downregulated (**Fig. 3H**).

### QKI represses ccRCC development by regulating the expression of HIF-1α and inhibiting angiogenesis

**Figure 4:**
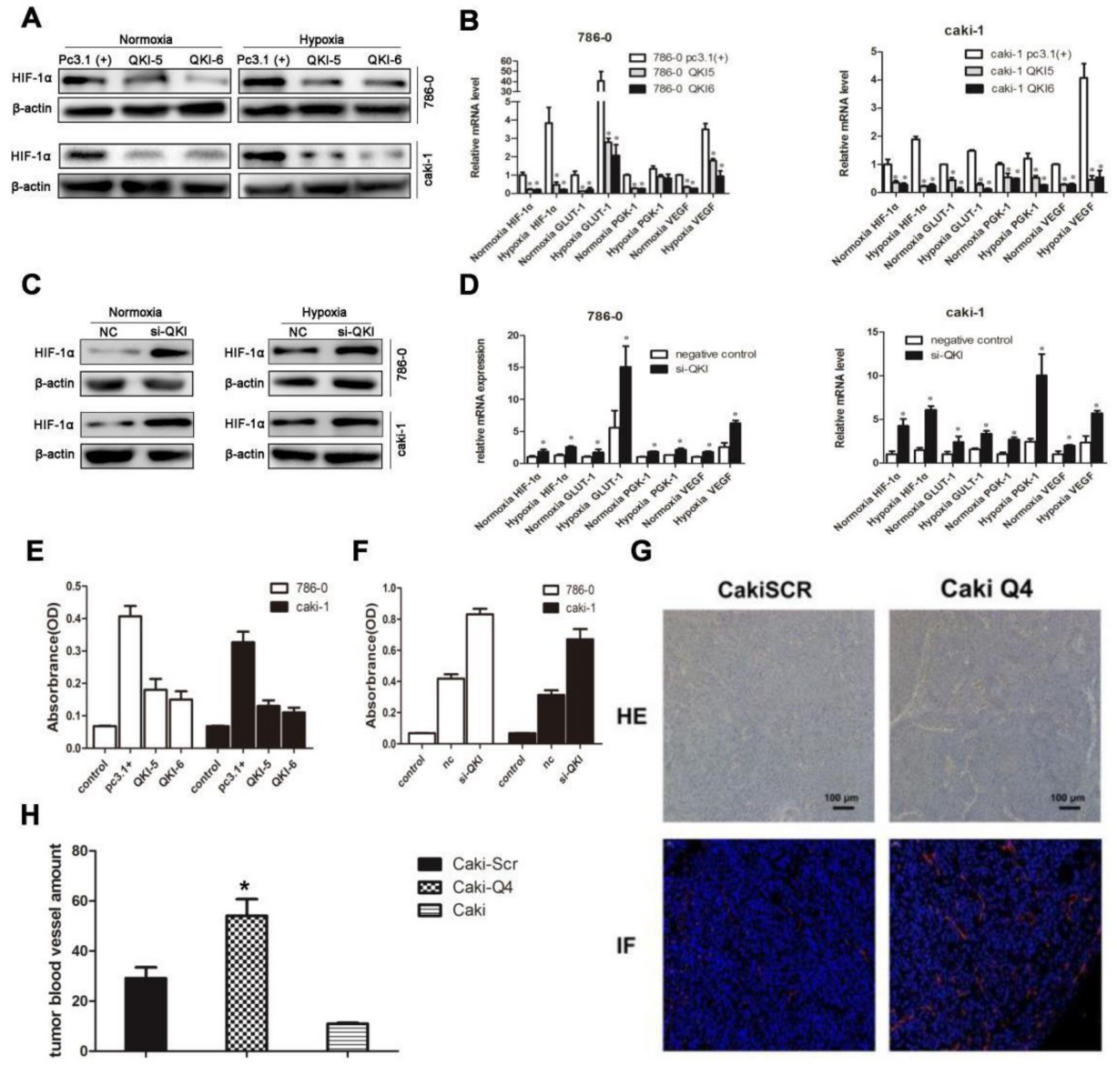
** QKI represses ccRCC development by regulating the expression of HIF-1α and inhibiting angiogenesis.** RT-PCR analysis showed that in the QKI overexpressing 786-0 **(A)** and caki-1 **(B)** cells, the HIF-1α,VEGF, GLUT-1, PGK-1 expression level was downregulated ,whereas in the QKI knock-down 786-0 **(C)** and caki-1 **(D)** cells, the HIF-1α, VEGF, GLUT-1, PGK-1 expression level was upregulated, the HIF-1α,VEGF, GLUT-1, PGK-1 expression level was even higher in hypoxia condition compare to normoxia condition. **(E)** VEGF protein level on QKI overexpression 786-0 and caki-1 cells. **(F)** VEGF protein level on QKI knockdown 786-0 and caki-1 cells. **(G)** HE staining (upper panel) and immunofluorescence(lower panel) of CD31 in nude-mouse transplanted tumor model (200×). **(H)** Microvessel count (MVC) of tumor mass. The data are presented as the mean ± SD and one -way ANOVA analysis for three independent experiments. * *P* < 0.05.

To further investigate the downstream targets of QKI in ccRCC, we scrutinized the coding and noncoding regions of transcriptional factors, which may contain potential QREs. Based on the screening results, HIF-1α was a potential candidate. Since the expression ofHIF-1α is also regulated by the oxygen concentration, we investigated normoxic and hypoxic group transfected with the QKI vectors. After 48 hours of culture, the QKI expression was upregulated and downregulated, respectively, in both the 786-0 and caki-1 cells. RT-PCR and western blot analysis showed that in the QKI-overexpressing 786-0 and caki-1cells, HIF-1α expression was downregulated, which was more obvious under hypoxic conditions (**Fig. 4A and B**). By contrast, in the 786-0 and caki-1 cells with QKI knockdown, the HIF-1α expression level was upregulated, and similar to the tendency mentioned above, the HIF-1α expression level was even higher under hypoxic than under normoxic conditions (**Fig. 4C and D**). To investigate the regulation of the downstream targets of HIF-1α, we tested vascular endothelial growth factor (VEGF), Glucose transporter 1(GLUT-1) and phosphoglycerate kinase-1(PGK-1), which play roles in angiogenesis, glycolysis, and glucose transport, respectively. To determine the relationship between these genes and the expression of QKI in ccRCC under both normoxic and hypoxic conditions, we performed qRT-PCR analysis. The data showed that in the QKI-overexpressing ccRCC cells, the VEGF, GLUT-1, and PGK-1 expression levels were downregulated, and even more so under hypoxic conditions (**Fig. 4B**). Conversely, in the QKI-knockdown ccRCC cells, the expression levels of these genes were upregulated. Moreover, the VEGF, GLUT-1, and PGK-1 expression levels were even higher under hypoxic conditions (**Fig. 4D**).

To further explore the effects of QKI up-and downregulation on angiogenesis, we used ELISA. Firstly, si-QKI small-interfering RNA and the QKI vector were used to transfect 786-0 and caki-1 cells for 24 hours. Additionally, the serum-containing medium was replaced with serum-free medium, and the cells were cultured for another 24 hours before the supernatants were harvested. After 48 hours post-transfection, the supernatants of 786-0 and caki-1 cells were harvested for ELISA. The assay showed that VEGF expression was downregulated in the QKI-overexpressing groups (**Fig. 4E**) and conversely was upregulated in the knockdown groups. Again displaying a similar tendency to the one mentioned above, the VEGF expression level was even higher under hypoxic, as compared to normoxic conditions (**Fig. 4F**).To study whether QKI would affect angiogenesis *in vivo*, we injected normal caki-1 cells and QKI-knockdown caki-1 cells into the dorsal area of nude mice. We conducted HE-staining and immunofluorescence staining for CD31 in the tumor mass, which showed a significant increase of vascularization in the QKI knockdown group (**Fig. 4G**). Moreover, the MVC further corroborated this observation (**Fig. 4H**).

**Figure 5:**
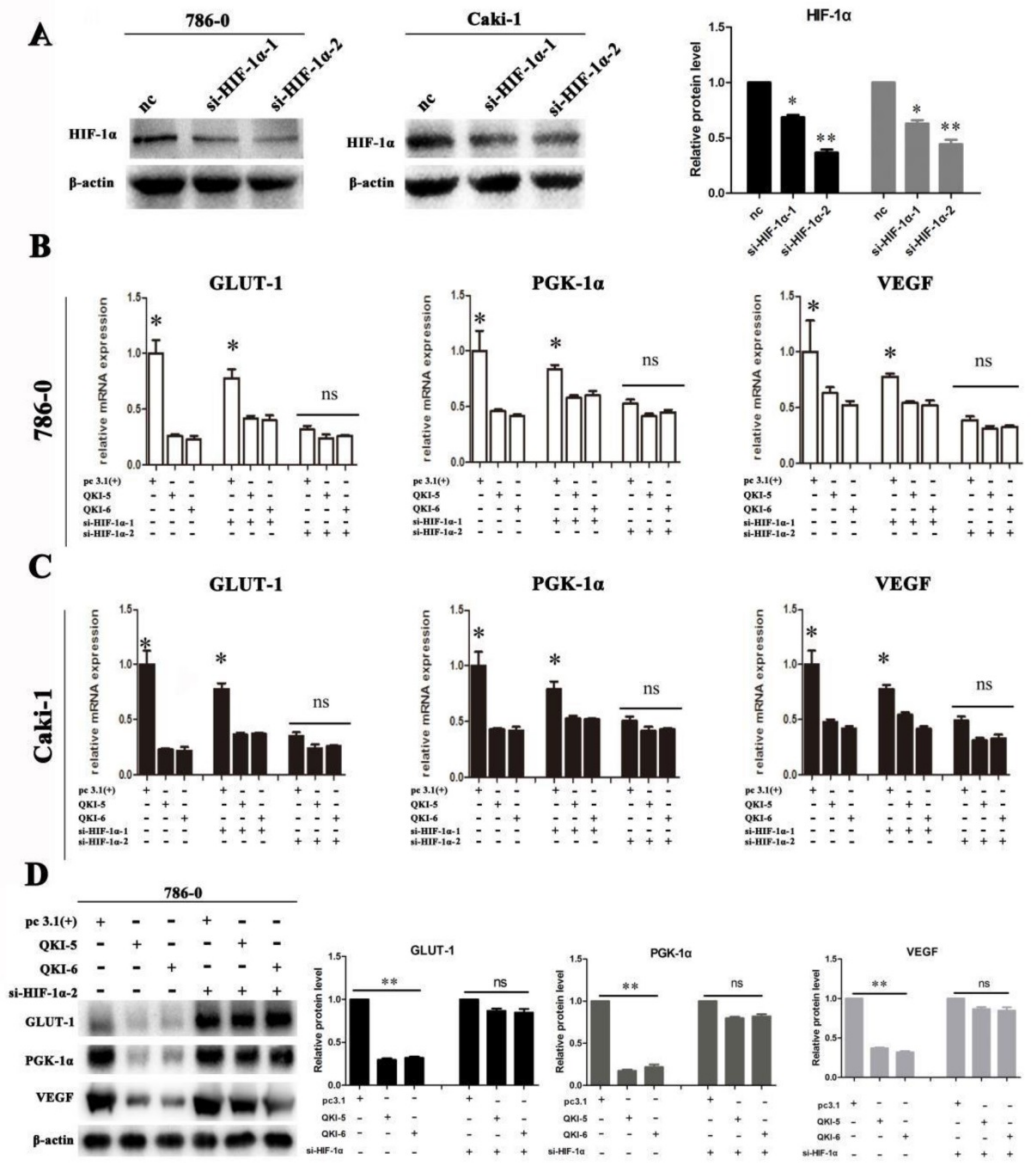
** Downregulation of HIF effects cell metabolism in ccRCC. (A)** Silence efficiency of small interfere RNA toward HIF-1α in 786-0 cell line. Co-transfection effect of GLUT-1,PGK-1,VEGF in 786-0 cell line **(B)** and caki-1 cell line **(C)** are shown. The data are presented as the mean ± SD and one -way ANOVA analysis for three independent experiments. * *P* < 0.05.

We next investigated if QKI directly affected the expression of VEGF, GLUT-1, and PGK-1, or of its effect was realized via HIF-1α. Firstly, two small-interfering RNAs (siRNAs) which specifically silence HIF-1α were designed and verified in 786-0 and caki-1 cells (**Fig. 5A**). Subsequently, siRNA transfection was conducted in both 786-0 and caki-1 cells 24 hours prior to transfection with the QKI vectors. After a further 48 hours, the cells were harvested and RT-PCR analysis was conducted. As shown in **Figure 5B and C**, when si-HIF-1α and the QKI vector were used to co-transfect the cells, them RNA expression levels of HIF-1α, as well as the bona-fide downstream genes VEGF, GLUT-1, and PGK-1 were not significantly downregulated compared to the control group. It can therefore be concluded that the mechanism by which the RNA-binding protein QKI was able to negatively regulate the expression levels of HIF-1α and its target genes VEGF, GLUT-1 and PGK-1 requires the expression of HIF-1α. The similar trend was verified in protein level in 786-0 cells by western blot assay (**Fig. 5D**).

### *In vivo* growth inhibition

**Figure 6:**
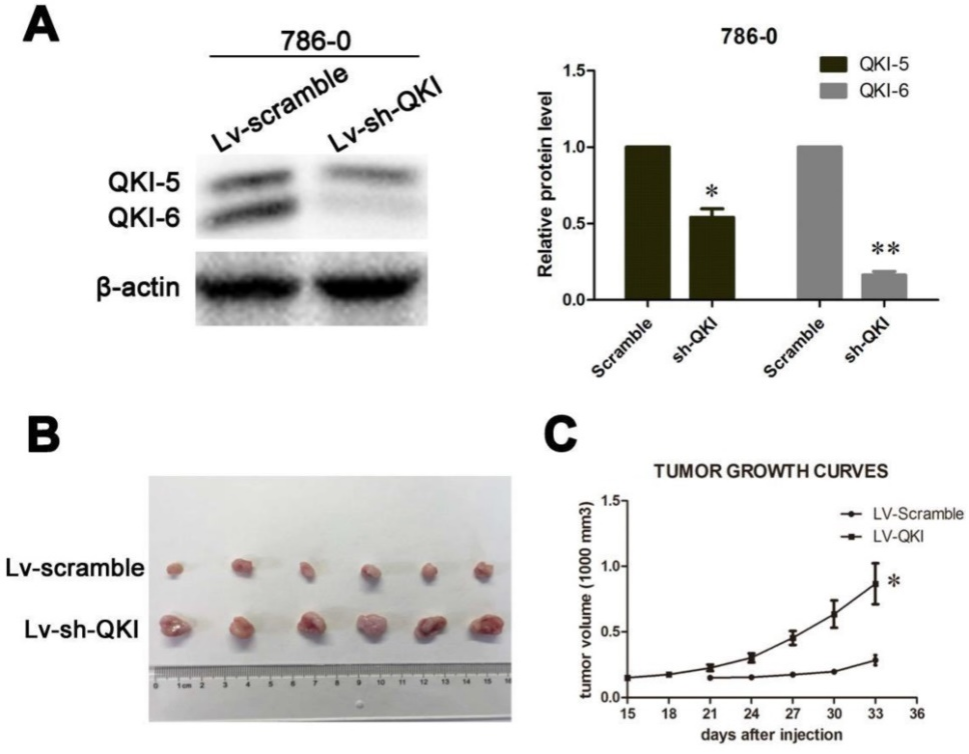
** QKI inhibits ccRCC growth *in vivo.* (A)** The efficiency of QKI knockdown in 786-0 cell line.** (B)** Images of excised tumors from twelve nude mice at 33 days after injection with vector-transfected cells and QKI-sh-transfected cells. **(C)** Tumor volumes were measured every three days. Statistical significance was evaluated with one-way ANOVA analysis,* *P* < 0.05.

After studying the role of QKI *in vitro,* we wanted to confirm the inhibitory role of QKI in tumor initiation *in vivo*. To do so, LV-QKI lentivirus particles were used to infect 786-0 cells, and the efficiency of transformation was verified as shown in **Figure 6A**. The resulting transformed cells were injected into nude mice, which were monitored for tumor formation every 3 days. As shown in **Figure 6B and 6C**, the tumors that grew in the LV-Scramble group were smaller than those in the LV-QKI group. In conclusion, LV-QKI 786-0 cells were more tumorigenic than the LV-Scramble 786-0 cells.

### Correlation of QKI expression with the clinical pathological features of clear cell renal cell carcinoma

To analyze the correlation of the QKI expression with different clinical factors, we conducted semi-quantitative analysis and divided the tissue samples into two QKI expression groups according to the QKI immunohistochemistry results: increased (> 25%) and decreased (<= 25%). The correlation of QKI expression levels in the ccRCC samples with different clinicopathologic factors is shown in **Table 4**. The QKI expression level was closely related with the Fuhrman nuclear grading and the intensity of dye (*P* < 0.05). Statistically significant correlations were not found between QKI and depth of invasion, pTNM stage, age at diagnosis, gender, primary tumor location and maximum tumor diameter (P>0.05) (**Table 4**).The coefficients of correlation between QKI and clinical pathological characteristics are shown in **Table 5**.

### Correlation of QKI expression with the survival of patients with clear cell renal cell carcinoma

**Figure 7:**
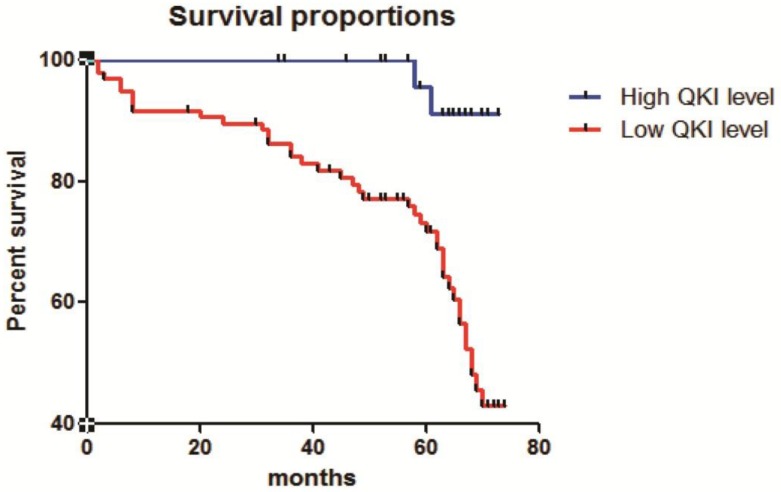
** Correlation of QKI expression with the clinical pathological features of clear cell renal cell carcinoma.** Kaplan-Meier overall survival curves for all 210 patients with kidney cancer stratified by high and low expression of QKI. Statistical significance was evaluated with one-way ANOVA analysis,* *P* < 0.05.

The correlation of QKI expression with patient survival was determined using the Kaplan-Meier estimator (**Fig. 7**). These results showed that decreased QKI expression was significantly correlated with poor survival. In the group showing non-decreasing QKI expression, the median survival time of clear cell renal cell carcinoma patients was undefined. However, in the decreased-QKI-expression group, the median survival time was only 68 months. The age at diagnosis, depth of invasion, TNM stage, gender, primary tumor location and maximum tumor diameter were not correlated with the survival time.

## Discussion

The incidence of renal cell carcinoma (RCC) accounts the sixth place in male patient and the ninth place in female patient 2, 3, 18. Despite of common treatments to renal cancer patients such as surgical resection and adjuvant therapy, the improvement of overall survival rate is still required. Traditional cytokine immunotherapy for advanced RCC can provoke an immune response against RCC, resulting in lasting, complete remission. Although current therapeutic means, including interferon α (IFN-α), low-dose interleukin 2 (IL-2), and high-dose IL-2. In particular, high-dose IL-2 therapy, effectively cure kidney cancer, it only works in approximately 10% of patients 19, 20. Therefore, the novel insight of biological therapies for clear cell renal cell carcinoma is urgently needed.

The incidence and development of renal cell carcinoma depends upon the expression of several oncogenes, such as HIF-1α, VEGF *et al.* 21. HIF-1α is frequently upregulated in renal cancer, which might be correlated with poor prognosis 22. HIF-1α acts as a downstream of epidermal growth factor receptor (EGFR). Thus, the inhibition of HIF-1α expression may become a promising approach to treat renal cancer.

The RNA-binding protein QKI is an important factor that attracts researchers' attention for its key role in tumor cell initiation and differentiation in various studies. Our previous studies demonstrated significant role of QKI in cancers 14-17. Here we showed that QKI plays similar roles in clear cell renal cell carcinoma as in other malignant tumors 14-17. Our study showed a decreased or even completely absent QKI expression in human ccRCC tissues and cell lines. Using antibodies specific for each carboxyl tail, Hardy *et al*. showed that in the mouse brain, all three isoforms are expressed in glial cells. Notably, QKI-5 locates in nuclear whereas QKI-6 and -7 retentate in cytoplasmic 23. Our results were highly consistent with the previous results, since QKI was mainly expressed in the nuclei of ccRCC, whereas in normal tissues QKI was expressed both in the nucleus and the cytoplasm. Western blot, MTT and FACS assays were conducted to test the inhibitory role of QKI in ccRCC. *In vitro*, the overexpression of QKI inhibited the proliferation of the 786-0 and caki-1 cell lines, blocked the cells' entry into the S phase, and promoted cell apoptosis. Using small interfering RNA, we confirmed the role of QKI in ccRCC. *In vivo*, the knockdown of QKI promoted tumor growth and upregulated the expression of HIF-1α. These data demonstrated that QKI plays a tumor-suppressive role in ccRCC.

QKI regulates the mRNA expression of many target genes at the posttranscriptional level. In fact, the expressions of hundreds of genes are regulated by direct binding of QKI at their 3′UTR or CDS regions harboring QRE-binding sites with a specific AYUAAY sequence 24, 25. Research showed that HIF-1α may have a potential QRE site in its CDS region, which means that HIF-1α may indeed be regulated by QKI 13. Judging from the data presented here, overexpressing QKI both under normoxic and hypoxic conditions can suppress the expression of HIF-1α and its downstream target genesin786-0 and caki-1 cells. Conversely, knockdown of QKI can promote the expression of HIF-1α and its downstream targets in these cells. When si-HIF-1α and QKI vector were used to co-transfect 786-0 and caki-1 cells, QKI was incapable of controlling the downstream targets of HIF-1α.

Combining the *in vitro* and *in vivo* data, our results strongly support the idea that QKI can inhibit the development of ccRCC by regulating HIF-1α and its downstream target genes, and that it requires the expression of HIF-1α. Clear cell renal cell carcinoma is associated with loss-of-function mutations in the VHL(von Hippel- Lindau) tumor suppressor gene. VEGF is an important target of HIF and a known potent mediator of angiogenesis 26. According to the results of ELISA, overexpressing QKI can suppress the expression of VEGF in 786-0 and caki-1 cells under both anoxic and normoxic conditions. Conversely, QKI knockdown can promote VEGF expression in these cells. These results mean that QKI may be used as a predictor in targeted therapy of ccRCC. Furthermore, QKI expression was closely correlated with Fuhrman nuclear grading, and our study also revealed a notable correlation of decreased QKI expression with a low survival rate, which indicated that QKI plays a key role in the development and prognosis of clear cell renal cell carcinoma.

Our previous reports have shown that QKI functions as a suppressor of carcinogenesis in diverse tumors through the coordinated targeting multiple genes cell growth and differentiation genes 14-17. Although surgical resection is an important treatment mean for most patients with localized RCC, however, the prognosis is still poor in advanced RCC patients, or recurrent cases after surgery. It is therefore urgently needed to develop appropriate targeted therapies and matched predictors to confirm the efficacy of ccRCC treatment. In our study, we have demonstrated that QKI negative controls ccRCC cell proliferation and cell cycle progression, and a positive control in apoptosis. Further elucidating the molecular mechanisms of QKI regulation may provide new avenues for ccRCC therapy. Additionally, the decrease of QKI expression is closely correlated with Fuhrman nuclear grading and poor survival. Our study thus revealed new regulation patterns of QKI and may provide a new diagnostic index as well as a novel target for ccRCC therapy.

## Figures and Tables

**Table 1:** Expression of QKI in Adjacent normal tissues and Clear cell renal cell carcinoma (ccRcc) tissues.

Group	QKI(-)	QKI(+)		
ccRcc	41	120		
Normal	4	157	*Χ^2^*=20.99	*p*<0.005

**Table 2:** QKI nuclear positive expression in Adjacent normal tissues and Clear cell renal cell carcinoma (ccRcc) tissues.

Group	Nuclear +	Nuclear -		
ccRcc	121	40		
Normal	122	39	*χ2*=0.07	*p*>0.005

**Table 3:** QKI cytoplasm positive expression in Adjacent normal tissues and Clear cell renal cell carcinoma (ccRcc) tissues.

Group	Cytoplasm +	Cytoplasm -		
ccRcc	10	151		
Normal	125	36	*χ2*=84.34	*p*<0.001

**Table 4:** Correlation of QKI with clinicopathologic characteristics of patients with ccRcc.

Variable	N	QKI expression		QKI expression		P
		Low (<=25%)	%	High (>25%)	%	
**pathology**						P=0.03
Ⅰ	34	16	47	18	53	
Ⅱ	74	47	64	27	36	
Ⅲ	44	35	80	9	20	
Ⅳ	9	6	67	3	33	
**pTNM stage**						P=0.378
Ⅰ	95	58	61	37	39	
Ⅱ	42	30	71	12	29	
Ⅲ	21	13	62	8	38	
Ⅳ	3	3	100	0	0	
**Intensity of dye**						P=0.001
Negative	41	33	80	8	20	
Weakly positive	42	34	81	8	19	
Positive	28	15	54	13	46	
**Gender**						P=0.633
Male	100	66	66	34	34	
Female	61	38	62	23	38	

**Table 5:** Correlation coefficients of QKI with clinicopathological characteristics ccRcc.

Variable	Correlation coefficient (r)	P value
Survival time	0.255	0.001
Sex	0.038	0.635
Age at diagnosis	-0.091	0.249
Maximum diameter	-0.115	0.147
Primary location	0.007	0.932
pTNM stage	-0.079	0.298
Depth of invasion	-0.035	0.643
Pathology grade	-0.203	0.006

## Abbreviations

ccRCC: clear cell renal cell carcinoma
EGFR: epidermal growth factor receptor
ELISA: Enzyme-linked immunosorbent assay
HIF-1α: hypoxia-induced factor-1a
MTT: 3-(4, 5-methylthiazol-2-yl)-2, 5-diphenyl-tetrazolium bromide
MVC: microvessel count
PGK-1: phosphoglycerate kinase-1
QRE: QKI responsive elements
RCC: renal-cell carcinoma
VHL: Von Hippel-Lindau
